# Characterizing microbial diversity and metabolic pathways in yak milk and fermented yak milk based on metagenomics: A study from Ganzi Tibetan autonomous prefecture

**DOI:** 10.1016/j.fochx.2025.102198

**Published:** 2025-01-18

**Authors:** Jie Zhang, Yangbo Jiao, Kaiyang Liu, Wenyou Situ, Bilige Menghe, Yongfu Chen, Musu Zha

**Affiliations:** a Key Laboratory of Dairy Biotechnology and Engineering, Ministry of Education, Inner Mongolia Agricultural University, Hohhot, Inner Mongolia 010018, China; b Inner Mongolia Key Laboratory of Dairy Biotechnology and Engineering, Hohhot, Inner Mongolia 010018, China; c National Center of Technology Innovation for Dairy, Hohhot, Inner Mongolia 010080, China

**Keywords:** Yak milk, Natural fermentation, Shotgun metagenomics sequencing technology, Microbial diversity and pathway

## Abstract

Kangding and Litang are the capital and pivotal county respectively within Ganzi. The region's distinctive geographical and climatic environment has endowed yak milk (YM) and its products with unique microbial resources, which play a crucial role in product quality and flavor. Therefore, it is important to understand their microbiota. We analyzed microbiota and metabolic pathways in YM. Results revealed 207 species, with *Pseudomonas* unclassified, *Acinetobacter johnsonii* dominant in YM, and *Lactobacillus delbrueckii*, *Streptococcus thermophilus* in fermented yak milk (FYM). YM exhibited lower microbial and bacteriophage diversity. Bacteriophage diversity was primarily targeting harmful microbes. Yak and camel milk showed similarities, while koumiss and fermented camel milk shared dominant bacteria. Metabolic pathways in YM were enriched with carbohydrates, amino acids, fats, and purine metabolism. In conclusion, this study provides information on the microbial resources and related metabolic pathways in yak milk and naturally fermented yak milk in the Ganzi region of China.

## Highlights

• Revealed microbiota in yak milk from Ganzi via Shotgun metagenomic sequencing.
• Comparison of microbial composition and metabolic pathways in Kangding and Litang.
• The dominant bacterial species were L. *delbrueckii* and *S. thermophilus* after fermented.
• Bacteriophages in YM primarily targeted harmful microbes.
• Metabolic pathways differed in carbohydrates, amino acids, and fats.

## 1 Introduction

The yak (*Bos grunniens*) is a long-haired bovine species that represents the primary livestock breed in the Qinghai-Tibet Plateau (Cui et al., 2016). This prominence is attributed to the adaptability of the yak to harsh environmental conditions, including extreme cold, high altitude, and low oxygen levels. Yaks are multipurpose animals that serve multiple purposes, providing meat and dairy products (Xiong et al., 2023), of which yak milk (YM) is one of the most valuable. YM contains higher concentrations of proteins, essential amino acids, lactose, minerals, and vitamins than conventional bovine milk. Fermented yak milk (FYM), also known as *kurut*, is a traditional fermented dairy product produced from YM. The FYM was manufactured without the addition of commercial starter cultures or preservatives. Its surface is characterized by yellow, firm milk skin, whereas the interior remains pure white and viscous. The production of FYM spans thousands of years, with the development of a complex microbial ecosystem dominated by lactic acid bacteria (LAB) (Jiang et al., 2014).

The unique geographical characteristics of the Ganzi region, coupled with the preservation of traditional production techniques over millennia, have endowed the FYM with an exceptional reservoir of microbial resources. Various factors, including temperature, altitude, and regional breeding environments, significantly influenced the microbial composition of YM and FYM. Ma et al. (2021) explored the microbial diversity of YM across different altitudes, revealing that *Firmicutes* and *Proteobacteria* were the dominant bacterial phyla in the YM of Qinghai. Furthermore, studies have reported regional variability in LAB composition in traditional FYM. Ding et al. (2022) analyzed the bacterial composition of traditional FYM in five major ecotypes of the Qinghai-Tibetan Plateau, identifying *Lactobacillus delbrueckii*, *Streptococcus salivarius*, and *Streptococcus thermophiles* as the predominant species. In addition to microbial communities, such as LAB, bacteriophages (viruses that infect bacteria) are also an important component of the microbial ecosystem in fermented dairy products, with bacteriophage infections often causing fermentation failures. Consequently, a comprehensive investigation of the microbial composition of FYM is vital to control the quality of fermented milk. Shotgun metagenomic sequencing technology significantly assists in analyzing the structural and functional characteristics of the microbiota in traditional fermented dairy products (Zheng et al., 2021). However, most prior studies on the FYM microbiota have relied on 16S rRNA high-throughput sequencing analysis rather than shotgun metagenomic approaches. Furthermore, studies examining bacteriophages and different milk-derived microorganisms are scarce. It is imperative to judiciously employ metagenomic technologies to advance the understanding of microbial communities and metabolic pathways in fermented dairy products such as FYM. These efforts will provide robust theoretical insights into the microorganisms in traditional fermented milk originating from different sources.

This study collected YM and FYM samples from two regions within the Ganzi Tibetan Autonomous Prefecture, Sichuan Province. Kangding serves as the seat of government and the political and cultural center of Ganzi Prefecture. At the same time, Litang is an important hub in southern Ganzi, known for its rich Kham culture and grassland ecology. Shotgun metagenomic sequencing technology was used to analyze the microbial composition, bacteriophages, and functional characteristics of the samples. Furthermore, we investigated microbial differences between milk and fermented milk derived from various animal sources. As a distinctive natural, ecological, and economic resource, YM has considerable potential for advancing the development and utilization of yak-related dairy products, which could enhance the growth of highland animal husbandry in China. The objective of this study is to provide a reference for the exploration and utilization of YM microbial resources.

## 2 Materials and methods

### 2.1 Sample collection

**Fig. 1:**
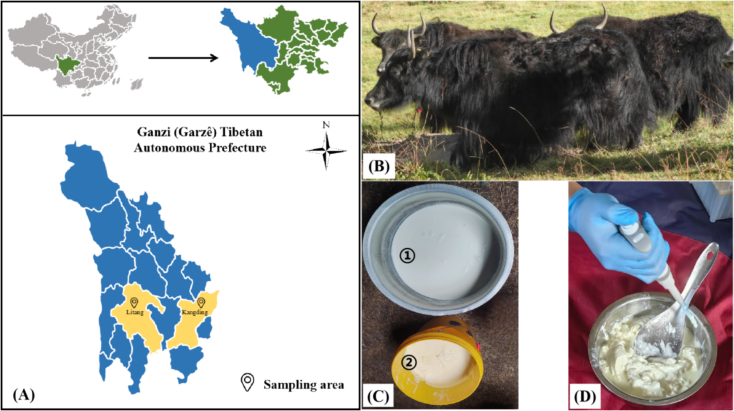
Locations of collecting the yak milk and fermented yak milk samples from Ganzi region, China (A). Photos of yaks in Ganzi (B). Photos of yak milk and fermented yak milk at the sampling area. ① shows yak milk, and ② shows fermented yak milk (C). Sampling diagram (D).

A total of 15 YM and 15 FYM samples were collected from villages in Kangding and Litang, Ganzi (Garzê) Tibetan Autonomous Prefecture, Sichuan Province, China. To ensure consistency, both YM and their corresponding FYM were obtained from the same herder households. The sampling areas were situated at altitudes ranging from 3650 to 3950 m, with latitudes ranging from 29°56′ N to 30°18′ N and longitudes from 99°46′ E to 101°33′ E, as detailed in Supplementary Table 1 (Table S1) and Fig. 1. Samples were classified into four distinct groups: YM from Litang (LT-YM), FYM from Litang (LT-FYM), YM from Kangding (KD-YM), and FYM from Kangding (KD-FYM).

All herder households used traditional natural fermentation methods to produce FYM following the method described by Dong et al. (2003). After collection, all samples were transferred to sterile enzyme-free centrifuge tubes. The samples were then rapidly frozen in liquid nitrogen and transported to the Key Laboratory of Dairy Biotechnology and Engineering at Inner Mongolia Agricultural University (Hohhot, China), where they were stored at −80 °C for subsequent metagenomic analysis.

### 2.2 DNA extraction

The YM and FYM samples were thawed at approximately 25 °C, and sample pretreatment was conducted according to the manufacturer's instructions. DNA was extracted using the DNeasy PowerFood Kit (Omega Bio-tek Inc., Norcross, GA, USA), a specialized kit designed to isolate inhibitor-free DNA from cultured food samples. This kit can produce high-quality DNA while efficiently removing PCR inhibitors, such as lipids and polysaccharides, from complex food matrices (Yang et al., 2021). The concentration, purity, and integrity of the extracted DNA were evaluated using a micro-UV spectrophotometer and 1 % agarose gel electrophoresis.

### 2.3 Metagenomic analysis

**Metagenomic sequencing and quality control.** After confirming the quality of DNA samples, they were randomly fragmented using an ultrasonic disruptor. The library preparation process encompassing end repair, A-tailing, adapter ligation, purification, and PCR amplification was subsequently performed. Upon completion of library construction, initial quantification was carried out using a Qubit 2.0 fluorometer, followed by dilution to the desired concentration. The insert size of the library was evaluated using an Agilent 2100 Bioanalyzer (Agilent, Santa Clara, CA, USA). Libraries meeting the predetermined insert size criteria were further assessed for effective concentration using real-time PCR to ensure quality (Cock et al., 2010).

Sequencing was conducted bidirectionally on an Illumina NovaSeq 6000 platform (Illumina, California, USA), generating paired-end reads of 150 bp in length. Reads were pre-processed using KneadData software (http://huttenhower.sph.harvard.edu/kneaddata; v0.7.5) and aligned against the bovine genome to eliminate host DNA sequences. High-quality reads that remained post-processed were retained for further analyses (Cock et al., 2010). Following the removal of low-quality sequences and host sequences (bovine genome), a total of 215.68 Gb of high-quality sequence data were obtained from 30 samples, with an average of 7.19 Gb per sample (Table S2).

**Metagenomic assembly.** High-quality Illumina metagenomic samples were assembled using MEGAHIT (v1.2.9, parameters: –presetsmeta-large–min-contig-len300) (Li et al., 2015). The assembly results were evaluated using QUAST (ver. 5.0.2).

**Bioinformatics analysis of bacteriophages.** Bacteriophage sequences were compared with known virus sequences using Blastn (v.2.10.1+) against the NT virus database, with results filtered at an e-value threshold of ≤1e-5. The gene abundance of bacteriophages was quantified by combining the comparison results with those of Salmon (v0.8.1) for more accurate quantification.

Raw mare milk (RMM) and koumiss (K) were retrieved from a publicly available database (accession number PRJNA646341). Camel milk datasets were derived from a previous study by our group (Su et al., 2024). Bactrian camel milk is abbreviated as BCM, and naturally fermented camel milk as NFCM. Mare milk was collected from the Xinjiang Uygur Autonomous Region, whereas camel milk was collected from the Alxa Region in Inner Mongolia.

### 2.4 Bioinformatic and statistical analysis

Quality-controlled sequences were taxonomically assigned at the species level using the MetaPhlAn2, HUMAnN2, and ChocoPhlAn pan-genome databases. These tools enabled the determination of the relative abundance of species and metabolic pathways within each sample. Microbial diversity and compositional differences were analyzed at the phylum, genus, and species levels, revealing significant variations among the four groups: LT-YM, LT-FYM, KD-YM, and KD-FYM. Comparative analyses of metabolic pathways across groups identified pathways with statistically significant differences. Furthermore, correlation analyses were performed to explore the relationships between these pathways and the microbiota exhibiting significant compositional differences.

**Statistical Analysis**: Statistical differences between the groups were analyzed using the Wilcoxon rank-sum test, Kruskal-Wallis test, and Dunn's test. These tests were applied to assess the differences in microbial composition and metabolic pathways under different conditions. Two-sample *t*-tests were performed using SAS software (v8.0, SAS Institute) to analyze altitude-related effects between Litang and Kangding. To visualize the microbiota, and metabolic pathways, Principal Coordinate Analysis (PCoA), R language (v4.3.1), and Gephi software (v0.10.1) were used. Linear Discriminant Analysis (LDA) was performed using the Linear Discriminant Analysis Effect Size (LEfSe) software to identify species differences between samples. The LDA threshold was set at 3.0 to determine statistically significant species differentiation.

## 3 Results

### 3.1 Metagenomic sequencing information

A total of 217.39 Gb of raw data was generated from 30 samples, with an average of 7.19 ± 0.72 Gb per sample. After quality control, 215.68 Gb (99.24 %) of clean data were retained. Notably, the percentage of high-quality sequences relative to the raw data exceeded 98 % in each library and the coverage across all samples was above 98 %. These metrics indicate that the data comprehensively and accurately represent the microbial community composition of YM and FYM (Table S2).

### 3.2 Analysis of microbial alpha and beta diversity

**Fig. 2:**
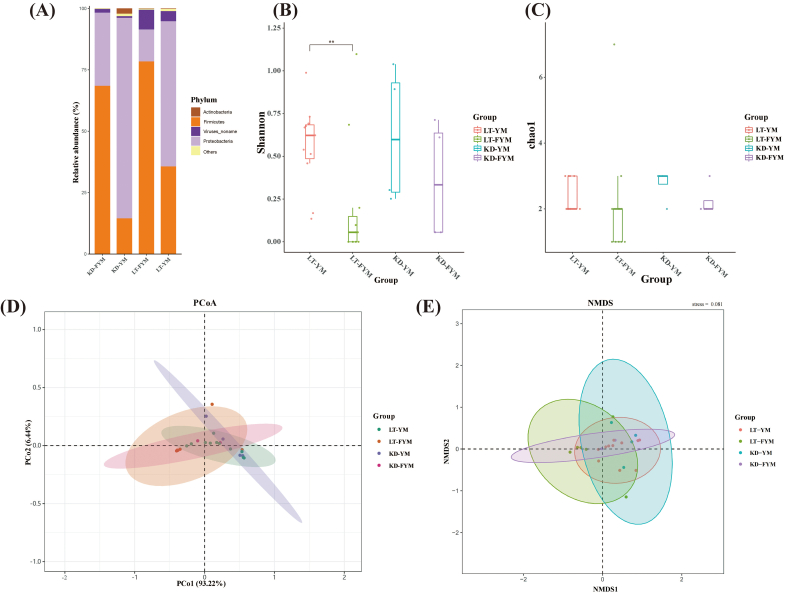
Relative abundances of microbiota in yak milk and fermented yak milk form different regions at the phylum level (A). α-diversity (B,C) and β-diversity (D,E).

**Fig. 3:**
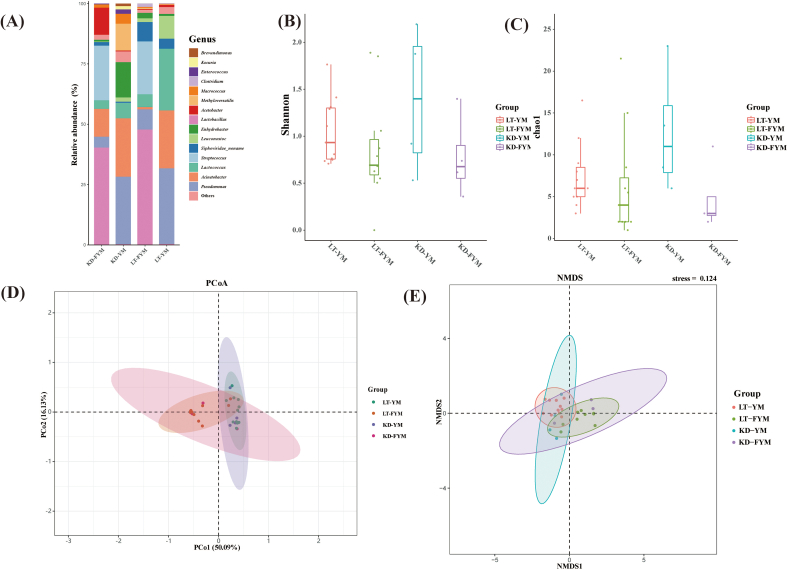
Relative abundances of microbiota in yak milk and fermented yak milk form different regions at the genus level (A). α-diversity (B,C) and β-diversity (D,E).

Metagenomic sequencing technology has been used to investigate microbial diversity, functional activity, and interactions with the environment. α-Diversity denotes the diversity of communities within a habitat, with richness and evenness serving as the two key indicators. The Shannon index measures both the richness and evenness of a community, whereas the Chao1 index is an indicator of species richness. In this study, metagenomic sequencing was employed to examine microbial community differences between YM and FYM in the Ganzi region. α-Diversity was analyzed at the phylum, genus, and species levels. The results revealed that natural fermentation of YM into FYM led to a decrease in the Shannon index at the phylum, genus, and species levels (Fig. 2B, 3B, 4D). Notably, in Litang, the Shannon index of FYM was significantly lower than that of YM at the phylum level (*P* < 0.05) and was significantly lower at the species level (*P* < 0.01). The results of the Chao1 index (Fig. 2C, 3C, and 4E) were comparable to the Shannon index. The Chao1 index of YM decreased after fermentation in different regions. Specifically, the Chao1 index of KD-FYM was significantly lower than that of KD-YM at the genus level (*P* < 0.05).

**Fig. 4:**
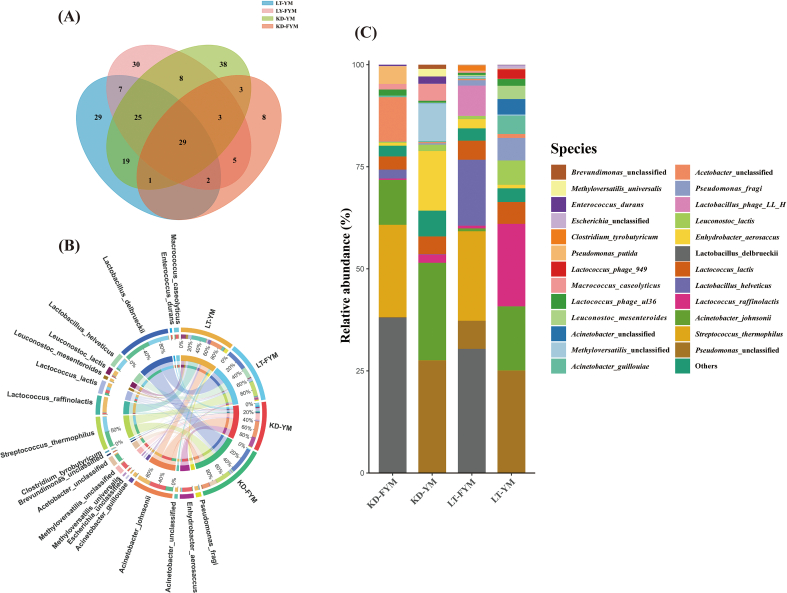

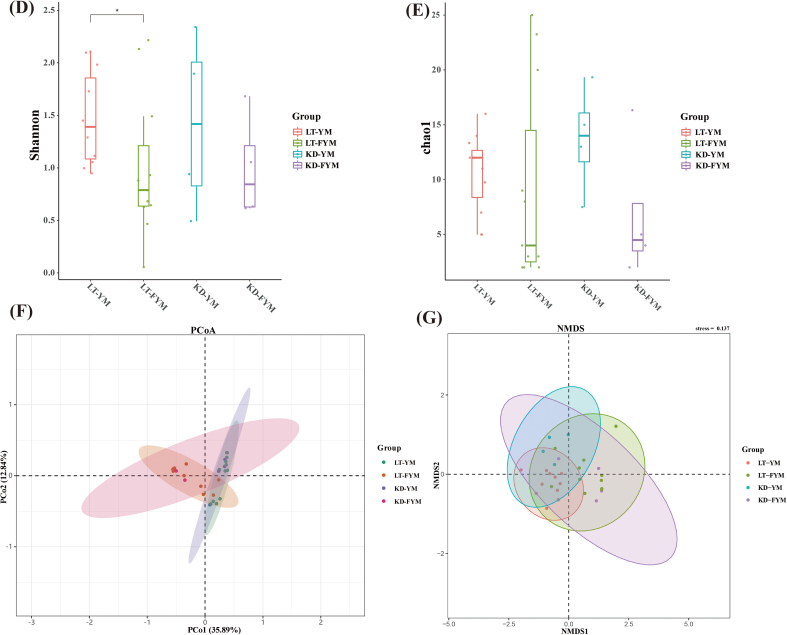
Microbial composition of yak milk and fermented yak milk form different regions at the species level. Venn analysis (A); Stacked bar chart of relative abundance (B); Circos plot (C); α-diversity (D,E) and β-diversity (F,G).

In this study, β-diversity was analyzed to compare the microbial compositions of YM and FYM across different regions. Principal Coordinates Analysis (PCoA) based on Bray-Curtis dissimilarities was used to assess the β-diversity among samples. At the phylum, genus, and species levels (Fig. 2D, 3D, 4F), YM, and FYM, demonstrated varying degrees of separation, particularly at the genus and species levels. However, a substantial overlap was observed among samples from different regions, suggesting that the microbial communities remained relatively homogeneous across the regions.

### 3.3 Analysis of microbial community composition

The microbial communities present in YM and FYM were analyzed at various taxonomic levels (Table S3). Fig. 2A illustrates the relative abundance of 11 phyla at the phylum level. In the YM samples from different regions, *Proteobacteria* and *Firmicutes* were the predominant phyla, collectively accounting for over 94.75 % of the microbial community. Similarly, in FYM, *Firmicutes* and *Proteobacteria* were the dominant phyla, with combined relative abundances exceeding 91.40 %. In both Litang and Kangding, the relative abundance of *Firmicutes* in FYM was higher than that in YM, and this trend was particularly pronounced in Litang.

Fig. 3A shows the relative abundance at the genus level, identifying 118 genera. In LT-YM, the three most abundant genera were *Pseudomonas* (31.30 %), *Lactococcus* (25.55 %), and *Acinetobacter* (24.02 %). After natural fermentation into FYM, the dominant genera were *Lactobacillus* (47.76 %), *Streptococcus* (21.92 %), and *Pseudomonas* (8.37 %). The results for samples from Litang were similar. In YM, *Pseudomonas* (27.96 %) was the most prevalent genus, whereas in FYM, *Lactobacillus* (40.29 %) and *Streptococcus* (22.62 %) were dominant. After fermentation, *Lactobacillus* emerged as the predominant genus in both regions, constituting more than 40 % of the microbial community.

At the species level, the Venn diagram (Fig. 4A) was used to assess species richness across different regions and samples. A total of 29 species were shared between YM and FYM, whereas 29 species were unique to LT-YM, 30 to LT-FYM, 38 to KD-YM, and 8 to KD-FYM. The results also indicated that species richness in FYM was lower than in YM, with KD-FYM exhibiting the lowest overall species richness. The relative abundance of these species is presented in Fig. 4B and C. In total, 207 species were identified in this study. The dominant species in LT-YM were *Lactococcus raffinolactis* (21.99 %), *P.* unclassified (21.15 %), and *A. johnsonii* (18.03 %). After fermentation, the three most prevalent species were *L. delbrueckii* (30.38 %), *S. thermophilus* (21.92 %), and *Lactobacillus helveticus* (16.16 %). Similarly, in KD-YM, the dominant species were *P.* unclassified (27.51 %), *A. johnsonii* (23.88 %), *Enhydrobacter aerosaccus* (14.60 %), which shifted post-fermentation to *L. delbrueckii* (38.05 %), *S. thermophilus* (22.62 %), *A. johnsonii* (10.99 %). In both Litang and Kangding, the fermentation process led to a notable shift in microbial dominance from potentially harmful microorganisms such as *A. johnsonii*, *P.* unclassified, *E. aerosaccus* to beneficial LAB, including *L. delbrueckii* and *S. thermophilus*. This microbial transition plays a critical role in the development of the distinctive texture and flavor of YM during fermentation.

### 3.4 Comparison of the microbial composition

Species-level analysis was conducted on microbial groups with relative abundances exceeding 1 % across different sample groups. The results presented in Fig. 7, Fig. 8 revealed significant variations among the four groups, with 10 species showing statistically significant differences (Fig. 7A). Among these, four species exhibited significant differences (*P* < 0.05), including *L. delbrueckii*, *Leuconostoc lactis*, *Leuconostoc mesenteroides*, and *Methyloversatilis universalis*, whereas six species, including *L. helveticus* and *Lc. Raffinolactis*, demonstrated highly significant differences (*P* < 0.01). Compared with YM, FYM contained a higher abundance of beneficial LABs, which are crucial for fermented milk flavor and quality. These LAB include L. *delbrueckii*, *L. helveticus*, and *S. thermophilus*. A comparison of LT-YM and LT-FYM (Fig. 7B) revealed that the relative abundances of L. *delbrueckii* and *S. thermophilus* in LT-FYM were significantly higher than those in LT-YM (*P* < 0.05). Similarly, a comparison between the LT-YM and KD-YM (Fig. 7C), indicates that the relative abundances of *Lc. raffinolactis* and *Leu. mesenteroides* was significantly higher in LT-YM than in KD-YM (*P* < 0.05).

To identify key microbiota with statistically significant differences across regions and sample groups, LEfSe was used to explore the key microbiota with statistical differences among all groups. An LDA value distribution histogram was also generated, displaying species with an LDA score greater than three (Fig. 8A, Fig. 8B). In this study, the microbiota enriched in LT-YM were (*s*) *Lc. raffinolactis*, (*s*) *Leu. Lactis* (*s*) *Leu. mesenteroides*, and (*s*) *P. fragi*; in LT-FYM, (*s*) *L. helveticus*; in KD-YM, (*s*) *M. universalis*, and in KD-FYM were (*s*) *L. delbrueckii*, and (*s*) *S. thermophilus*.

### 3.5 Analysis of bacteriophage composition

**Fig. 5:**
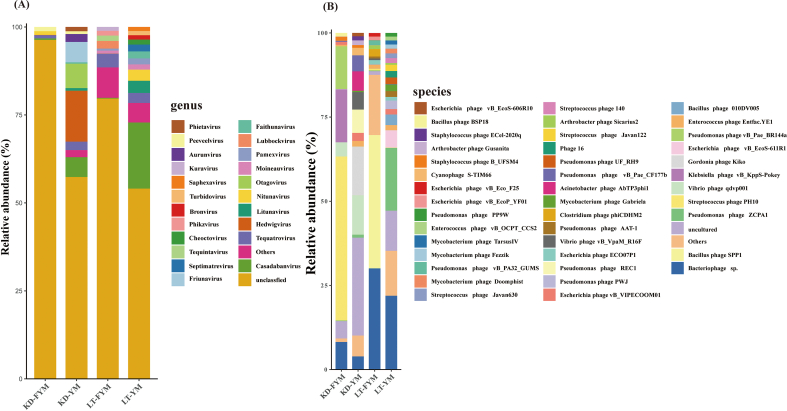

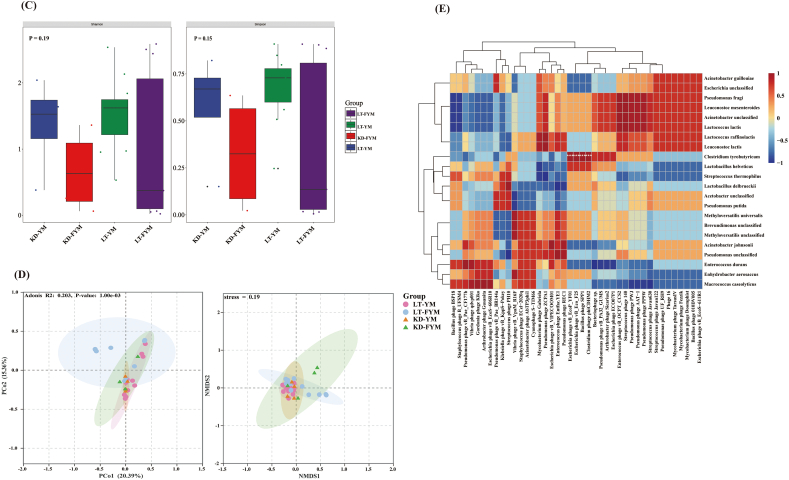
Analysis of phage in yak milk and fermented yak milk form different regions. Stacked bar chart of relative abundance at the genus and species level (A, B); α-diversity (C) and β-diversity (D); Heatmap of the correlation between differential phage and species based on Spearman's correlation analysis (E).

Bacteriophages, which are viruses that specifically infect bacteria, play a crucial role in fermented dairy products by directly influencing the fermentation process, product quality, and safety. To further explore their role, we investigated the bacteriophage composition in different samples, and the results are shown in Fig. 5. Owing to the limitations of the current bacteriophage databases, the number of annotated bacteriophages was relatively limited. At the genus level (Fig. 5A), the number of unclassified bacteriophages was higher in FYM than that in YM. After fermentation, the relative abundance of *Casadabanvirus* and *Litunavirus* decreased. At the species level (Fig. 5B), the relative abundance of *Enterococcus phage Entfac YE1* and *Escherichia phage vB VIPECOOM01* also declined after the fermentation of YM.

The α-diversity analysis at the species level (Fig. 5C) revealed no significant differences in Shannon and Simpson indices between the different regions and samples. However, both indices were consistently higher in YM than in FYM, indicating a greater bacteriophage diversity in YM. β-Diversity analysis (Fig. 5D) demonstrated a discernible separation trend between the bacteriophage compositions of YM and FYM, although substantial overlap was observed among samples from different regions. This finding suggests that fermentation alters bacteriophage composition, yet the compositions in YM and FYM across regions remain relatively similar, which is consistent with the results of microbial analysis.

To better understand the ecological interactions between bacteriophages and microbiota, we conducted a correlation analysis at the species level, focusing on bacteria with relative abundances greater than 1 %. The results (Fig. 5E) identified four significant positive correlations: *Clostridium tyrobutyricum* was positively associated with *Bacillus phage SPP1*, *Clostridium phage phiCDHM2*, *Escherichia phage vB EcoP YF01*, and *Escherichia phage vB Eco F25*.

### 3.6 Comparison of microbial composition in milk from different animal species

**Fig. 6:**
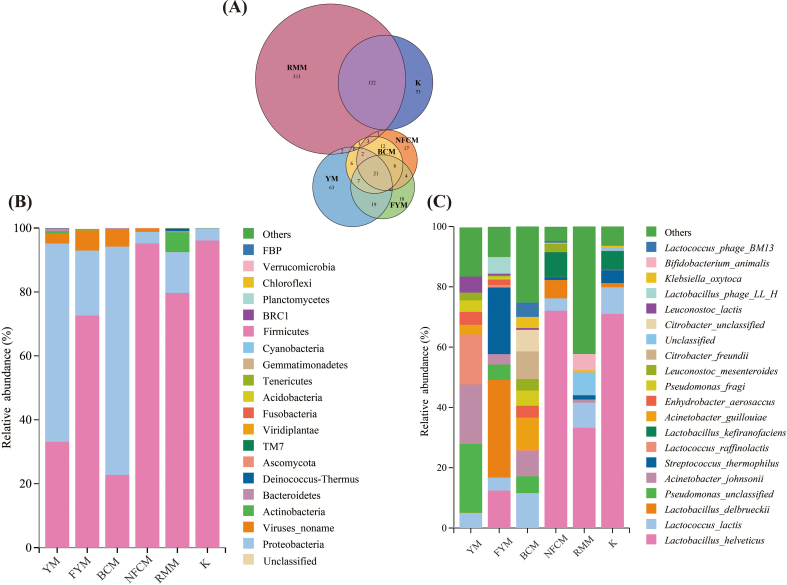

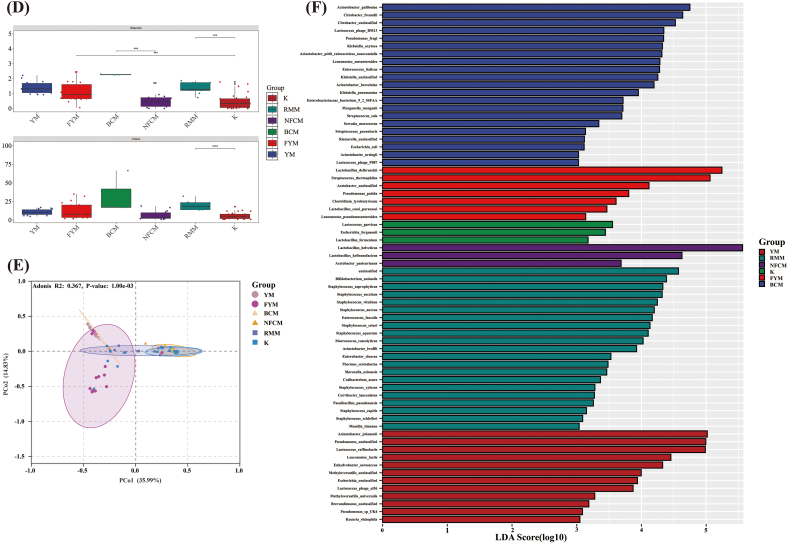
Analysis of microbial diversity and composition in different milk-derived matrices. Venn analysis showing shared and unique microbiota (A); Relative abundances of microbiota at the phylum (B) and species levels (C); α-diversity (D) and β-diversity (E); differences in microbiota at the species level among milk-derived matrices (F).

The microbial composition of milk from various animal sources, including mare milk and camel milk, was analyzed using public databases. Initially, a Venn diagram was constructed to examine species-level composition across the samples (Fig. 6A), revealing 17 species that were common to all samples. The results showed that the microbial composition of camel milk and mare milk was comparable to that of YM, with raw milk containing a greater number of bacterial species than fermented milk. The microbial composition at the phylum and species levels is presented in Fig. 6B and C. *Firmicutes* and *Proteobacteria* (relative abundance >92 %) were the predominant phyla in both raw and fermented milks. After fermentation, the relative abundance of *Firmicutes* increased significantly (relative abundance >72 %) across milk samples from different animal sources. In total, 761 species were identified, including 207 species in YM, 175 species in camel milk, and 551 species in raw mare milk (RMM). Before fermentation, the three most abundant species in Bactrian camel milk (BCM) were *Lc. lactis* (11.59 %), *A. johnsonii* (8.39 %), and *P.* unclassified (5.57 %), in RMM were *L. helveticus* (33.27 %), *Lc. lactis* (8.31 %), and *Bifidobacterium animalis* (5.37 %), and in YM were *P. unclassified* (22.84 %), *A. johnsonii* (19.60 %), and *Lc. raffinolactis* (16.66 %). Post-fermentation, the dominant species transitioned to *L. helveticus* (72.07 %), *Lactobacillus kefiranofaciens* (8.21 %) and *L. delbrueckii* (6.04 %) (NFCM); *L. helveticus* (71.04 %), *Lc. lactis* (8.78 %) and *L. kefiranofaciens* (6.26 %) (K); *L. delbrueckii* (32.43 %), *S. thermophilus* (22.10 %) and *L. helveticus* (12.44 %) (FYM) respectively. The results indicated a decrease in the abundance of potentially harmful microorganisms and dominance of beneficial LAB following milk fermentation. Notably, LAB diversity was higher in RMM than in the other milk types, and fewer species were associated with pathogenic bacteria.

**Fig. 7:**
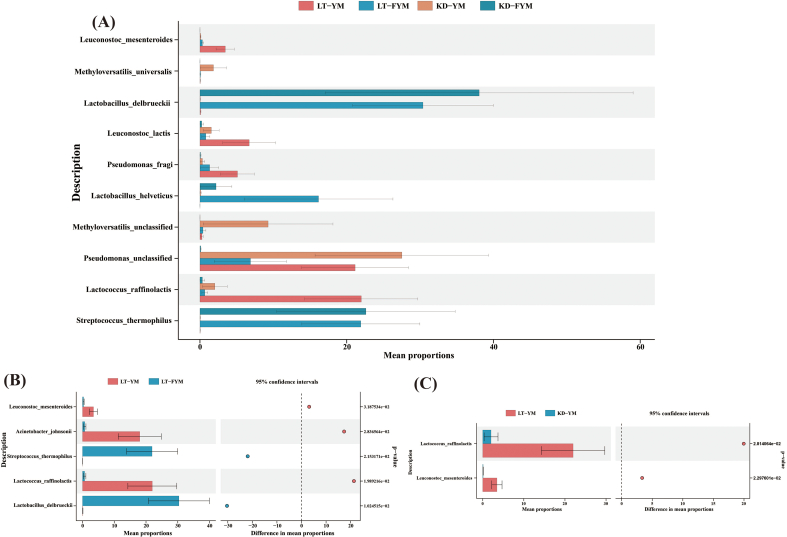
Analysis of microbial differences between yak milk and fermented yak milk from different regions at the species level (A). Differences between yak milk and fermented yak milk in Litang (B). Differences between Litang and Kangding of yak milk (C).

**Fig. 8:**
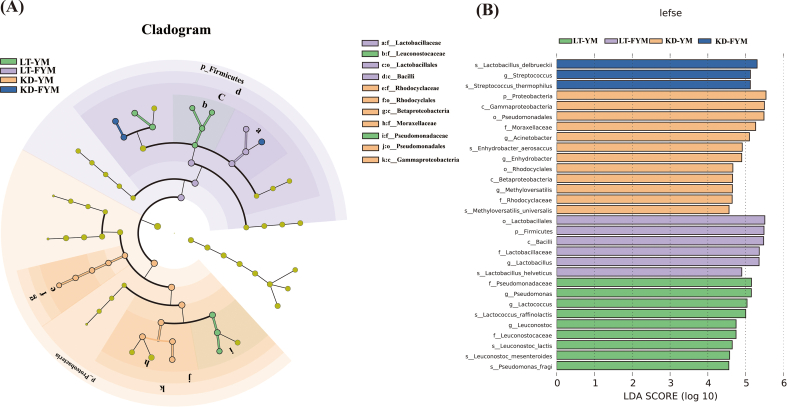
The LefSe analysis of microbiota between yak milk and fermented yak milk form different regions at the species level (A). The bacteria between yak milk and fermented yak milk. Statistically significant groups are reported with LDA scores (log10) >3. (B).

Next, we analyzed the α-diversity and β-diversity (Fig. 6D, Fig. 6E) in different samples at the species level and observed that the microbial diversity in different milk samples from animal sources in our study was higher than that in fermented milk, particularly camel milk and mare milk. The Shannon index of milk was significantly higher than that of fermented milk (*P* < 0.001). In addition, the Shannon index of YM was significantly higher than that of the RMM (*P* < 0.001). The Chao1 index results are consistent with those of the Shannon index. The Chao1 index of fermented milk decreased significantly, notably in mare milk (*P* < 0.0001). Principal Coordinates Analysis (PcoA) based on Bray-Curtis dissimilarity was employed to analyze the β-diversity among samples from different animal sources. At the species level, YM demonstrated a distinct separation trend from other milk types, whereas camel milk and mare milk exhibited a degree of overlap, suggesting that their microbiota were relatively similar.

Finally, Linear Discriminant Analysis Effect Size (LEfSe) was used to identify key groups with significant differences in microbial abundance among different samples. The results revealed that species with an LDA score greater than 3 (Fig. 6F). The bacterial groups enriched in YM were *P.* unclassified, *A. johnsonii,* and *Lc. raffinolactis*, in FYM were *L. delbrueckii*, *S. thermophilus*, in BCM were *A. guillouiae*, *Citrobacter freundii*, and in NFCM were *L. helveticus*, *L. kefiranofaciens* and *Acetobacter pasteurianus*; *B. animalis*, *Staphylococcus saprophyticus* and *Staphylococeus succinus* were enriched in RMM, whereas *Lactococcus garvieae*, *Escherichia fergusonii,* and *Lactobacillus fermentum* were enriched in K.

### 3.7 Analysis of the differences in the metabolic pathways

This study identified the metabolic pathways of microbial gene families using metagenomics, revealing a total of 472 metabolic pathways across all samples (Table S4). These bacteria-derived pathways were aligned with the microbial species composition results. As shown in Supplementary Fig. 1 A (Fig. S1A), differential pathway analysis across the four groups identified 78 pathways, including pyruvate fermentation to isobutanol, L-ornithine biosynthesis, and peptidoglycan biosynthesis III, which exhibited significant differences (*P* < 0.05). Additionally, 55 pathways, including L-tryptophan biosynthesis and CDP-diacylglycerol biosynthesis II, showed significant differences (*P* < 0.01). Notably, five pathways, including pyridoxal 5′-phosphate biosynthesis I, flavin biosynthesis III, the super pathway of heme biosynthesis from glutamate, tetrapyrrole biosynthesis I, and lipid IVA biosynthesis, showed extremely significant differences (*P* < 0.001). In summary, the majority of differential pathways were enriched in YM, whereas only a small number of pathways were enriched in FYM.

Next, we analyzed the differential pathways between LT-YM and LT-FYM. As illustrated in Fig. S1B, 74 pathways showed significant differences. Among these, lipid IVA biosynthesis and tetrapyrrole biosynthesis II were significantly different (*P* < 0.001). Furthermore, 23 pathways, including L-ornithine biosynthesis and phosphopantothenate biosynthesis I, showed significant differences (*P* < 0.01). Moreover, 49 pathways, including the C4 photosynthetic carbon assimilation cycle, showed significant differences (*P* < 0.05). Most of these pathways were enriched in YM. We then analyzed the differential pathways in KD-YM and KD-FYM (Fig. S1C). L-isoleucine biosynthesis IV was exclusively present in YM, resulting in its relative abundance being significantly higher than that in FYM (*P* < 0.05). Finally, we analyzed the differential pathways in FYM from different regions (Fig. S1D). Six pathways were significantly enriched in YM (*P* < 0.05), including the UDP-glucose-derived O-antigen building block biosynthesis and allantoin degradation pathways. Except for the two aforementioned differential pathways, the remaining four were observed exclusively in YM.

### 3.8 Correlation analysis of microbial communities and metabolic pathways

To investigate the interactions between the microorganisms identified in different regions and samples, species with a relative abundance exceeding 1 % were selected to establish an interaction network (Fig. S2A). The network exhibited a density of 0.122, an average degree of 2.3, and a diameter of 1, comprising 23 node connections and 20 nodes, of which 13 were positively correlated and 10 were negatively correlated. Most of the positive correlations associated with *Firmicutes* were observed with *Proteobacteria*, with a few correlations within *Firmicutes* and a positive correlation with *virus noname*. *Proteobacteria* exhibited both positive and negative correlations within phyla. Only one negative correlation was observed with other genera, specifically between *E. aerosaccus* and the *Lactococcus phage ul36*. Among *Firmicutes*, *L. delbrueckii*, *Leu. lactis*, and *Lc. raffinolactis* was negatively correlated with *P.* unclassified and *S. thermophilus*, respectively.

The differential pathways (*P* < 0.01) and species (relative abundance >1 %) in the four groups were further analyzed using Spearman's correlation analysis (Fig. S2B). *L. delbrueckii* and *S. thermophilus* exhibited negative correlations with the majority of pathways. Furthermore, *Pseudomonas putida* demonstrated negative correlations with PWY0–42:2-methylcitrate cycle I and PWY-7323: super pathway of GDP-mannose-derived O-antigen building block biosynthesis. The pathway POLYAMINSYN3-PWY: superpathway of polyamine biosynthesis II also displayed negative correlations with *Clostridium tyrobutyricum* and *Lactobacillus_phage_LL_H.* Only a limited number of microorganisms and metabolic pathways were positively correlated, such as *A.* unclassified, *P.* unclassified*,* PWY-5747:2-methylcitrate cycle II, and PYRIDNUCSAL-PWY: NAD salvage pathway I, among others.

## 4 Discussion

YM and related fermented dairy products are important to herders in the plateau regions. These areas typically exhibit relatively extreme ecosystems and severe climatic conditions that may harbor distinct microbial communities (Wu et al., 2009). Although research has been conducted on YM and FYM from plateau regions such as the Qinghai-Tibet Plateau, there is a scarcity of studies that simultaneously examine the microbial composition, dynamics, and metabolic pathways of YM and FYM. Therefore, using shotgun metagenomic sequencing technology, we conducted a comprehensive analysis of the microbial composition in YM and FYM from Kangding and Litang of Ganzi (Garzê) Tibetan Autonomous Prefecture, China, by examining the microbial communities. HUMAnN (V2.0) was used to analyze metabolic pathways.

Previous research has shown that microbial diversity in low-altitude areas with warm climates is higher than that in high-altitude areas with relatively cold climates (Ding et al., 2022). Our findings are consistent with these observations. The average altitude of the sampling area in Litang was 3848.26 m, whereas that in Kangding was 3765.32 m. The altitude of Kangding was significantly lower than that of Litang (*P* < 0.05). In addition, the annual average temperature in Litang is 3.0 °C, whereas that in Kangding is 7.1 °C. Analysis of α-diversity revealed that Kangding's YM and FYM indices were slightly higher than those of Litang. Beyond the impact of altitude on temperature, we speculate that Kangding's status as the administrative center of the Ganzi region may also play a role. The proximity of pastoral areas to residential zones, the broader range of herder activities, and the extensive interactions between people and livestock likely promote increased microbial diversity. After natural fermentation of YM, the richness of the microbiota decreased, and the Shannon index (at the phylum and species levels) decreased significantly (*P* < 0.05, *P* < 0.01). These findings are consistent with those of Guo et al. (2023) and Bao et al. (2022). This phenomenon occurred because the LAB in YM began to multiply in large quantities. As LAB became dominant, the microbial composition became singular and the diversity decreased. The β-diversity results indicated that the separation trend in YM and FYM (at the genus and species levels) was more pronounced, but there was substantial overlap in YM or FYM from different regions. This also demonstrates that the overall microbiota was relatively similar.

We conducted an in-depth analysis of the microorganisms across the four groups. The dominant genera in FYM of Litang and Kangding were *Lactobacillus* (47.76 % and 40.29 %, respectively) and *Streptococcus* (21.92 % and 22.62 %, respectively). Additionally, the relative abundance of *Lactobacillus* in Litang, which has a relatively high altitude, was higher than that in Kangding. This observation may be attributed to the higher acid tolerance of *Lactobacillus*. For instance, *Lactobacillus* demonstrates higher acid tolerance than *Streptococcus* and *Lactococcus*, which enables *Lactobacillus* to maintain higher fermentation activity during hydrolysis metabolism (Bao et al., 2012). The lower temperatures associated with Litang's higher altitude likely necessitate a longer fermentation period for FYM, which could explain the higher abundance of Lactobacillus in LT-FYM. The lower temperatures associated with Litang's higher altitude likely necessitate a longer fermentation period for FYM, which could explain the higher abundance of *Lactobacillus* in LT-FYM. Acid-tolerant lactic acid bacteria (LAB) such as *L. delbrueckii*, *L. helveticus*, and *S. thermophilus* were enriched in both LT-FYM and KD-FYM. These LAB species convert carbohydrates into lactic acid via glycolysis and produce flavor-enhancing compounds such as diacetyl and acetoin, particularly *Streptococcus* (Kaneko et al., 2014), which play a crucial role in the production of fermented milk. Lactic acid contributes significantly to the characteristic sour taste of fermented milk. These factors are beneficial to the safety and flavor profile of FYM (Hu et al., 2022). Despite these benefits, YM and FYM from Kangding and Litang contained species related to foodborne pathogens and environmental contaminants, such as *A. johnsonii*, unclassified *Acetobacter*, and unclassified *Pseudomonas*. These species are commonly found in milk, and may serve as potential contaminants (Gatti et al., 2014). After natural fermentation, the relative abundance of these species decreased, which was consistent with the results at the genus level. Nevertheless, it is imperative to improve the environmental conditions during the production process, guide herders to progressively enhance their production methods, and support their transition to modern, standardized practices. These approaches can effectively mitigate or prevent the presence of pathogens in fermented dairy products in pastoral regions (Bao, He, & Liu, 2022).

The susceptibility of numerous fermented lactic acid bacteria (LAB) to bacteriophage infections has become a prevalent issue in the dairy industry, resulting in fermentation failure and significant economic losses (de Melo et al., 2018). Consequently, a more comprehensive understanding of the bacteriophage associated with fermented milk and its history of infection is of significant interest (Qi et al., 2024). The bacteriophages identified in this study were exclusively members of the phylum *Caudoviricetes*, which are common in dairy products. The hosts of the bacteriophages were predominantly pathogenic bacteria such as *Escherichia* and *Pseudomonas*. According to previous reports (Pujato et al., 2014), certain long-tailed phages with nonretractable tails may originate from raw milk. The diversity of bacteriophages in raw milk is considered to be the primary vector for the introduction of bacteriophages into dairy products (Kleppen et al., 2011). In this study, the samples were sourced from pastoral areas. As the sanitary conditions of the production environment lack strict standards and cannot be controlled, YM is contaminated with harmful bacteria, resulting in the annotation of bacteriophages that use harmful bacteria as hosts. FYM is produced through natural fermentation, and the current bacteriophage database may be insufficient for coverage of LAB phages in non-industrial environments, failing to annotate LAB phages. After fermentation, the α-diversity of the bacteriophages decreased, which was consistent with the microbial results. The decrease in diversity and increase in LAB abundance after fermentation also contribute to the reduction in bacteriophage diversity. By elucidating the role of bacteriophages in fermentation systems, we can improve our ability to control and utilize bacteriophages and regulate the fermentation process.

China has a vast territory and a diverse ecological environment. Different regions have developed distinct livestock breeding practices, with animals, such as yaks, horses, and camels, adapting to various living conditions, including plateaus, grasslands, and deserts. These unique breeding practices contribute to the diversity of raw and fermented dairy products and foster a wide range of LAB resources. By comparing milk samples from animals with distinct regional characteristics (yaks, mares, and camels were selected for this study), it is possible to explore how living environments, physiology, and other factors influence dairy microorganisms. This comparison not only highlights the unique characteristics of yak milk, but also provides insights into the similarities and differences in the microbial composition of dairy products from different livestock species. Post-fermentation, the dominant phylum in fermented milk from different animal sources was *Firmicutes*, which aligns with previous research findings (Zhang et al., 2017). Further analysis of the microbiota in milk from different animal sources at the species level revealed that the relative abundance of potentially harmful microorganisms such as *A. johnsonii* and *P.* unclassified was higher in BCM and YM, while *L. helveticus*, *Lc. lactis* and other LABs were dominant in the RMM. This observation is consistent with the results of previous studies (Cai et al., 2022) and corroborates with the β-diversity results. The overlap between BCM and YM was greater, and they exhibited a separation trend from RMM, indicating that the microbiota of BCM and YM were more similar, but differed more significantly from RMM. This may be attributed, on one hand, to the well-controlled environmental sanitation conditions of the stables and mare's milk production. However, this may also be due to the frequent use of K as a starter for the production of fermented dairy products from RMM. It is hypothesized that this practice may have led to the enrichment of related bacteria in the production environment, allowing these bacteria to adapt to the environment over many years of production. In addition, *B. animalis* was one of the top three species in the RMM, distinguishing it from the other milk types. Pan et al. (2023) demonstrated that *Bifidobacterium animalis* subsp. *lactis* can enhance the volatile flavor substances and sensory quality of fermented milk. This may be because the living environment of mares affects the colonization and growth of *Bifidobacterium* in milk, which could contribute to the unique flavor qualities of K. Following fermentation, L. *helveticus* and *L. kefiranofaciens* emerged as the predominant species in K and NFCM, whereas *L. delbrueckii* and *S. thermophilus* were predominant in FYM. This observation was consistent with the β-diversity results. Regarding the variations in microorganisms present in raw milk and naturally fermented milk from diverse animal sources, it is hypothesized that these differences may be attributed to factors such as physiological characteristics, dietary habits, microbial environment, chemical composition of milk, and its ecological adaptability. From the perspective of physiological characteristics, horses, camels, and yaks exhibit distinct physiological and immunological characteristics. The physiological attributes of horses may be more conducive to the proliferation of *Lactobacillus* (e.g., *L. helveticus* and *L. kefiranofaciens*). Mare milk is characterized by low fat content and high lactose content. Lactose is the primary carbon and energy source for *Lactobacillus*, and a low-fat environment is favorable for *Lactobacillus* growth. Consequently, these factors contribute to the higher relative abundance of *L. helveticus* in raw and fermented mare milk than in milk from other sources. Finally, it is well known that *L. delbrueckii* and *S. thermophilus* are the main strains with favorable fermentation characteristics. Based on this, we speculated that naturally fermented yak milk is a potential source of strains with good fermentation characteristics. This has the potential to be used in the fermentation of dairy products and other foods, which is also of significance for increasing the added value of yak milk.

During the fermentation process, the metabolism of numerous complex compounds is associated with microorganisms, which obtain these compounds to acquire the energy and nutrients essential for their survival and reproduction (Shrestha et al., 2022). A total of 472 metabolic pathways were identified in the YM and FYM groups. The pathways exhibiting significant differences (*P* < 0.05) were predominantly related to carbohydrate metabolism, amino acid synthesis and metabolism, fat synthesis and metabolism, and purine anabolism, which is consistent with the findings of Liang et al. (2024). These pathways are closely associated with amino acid and lipid catabolism, suggesting that catabolism of lipids and amino acids in FYM may significantly influence product quality. Carbohydrate metabolism is typically linked to physiological activities such as microbial energy acquisition, growth, and development (Rizo et al., 2020). This holds considerable significance for the large-scale proliferation of LAB in milk and the formation of unique qualities in fermented milk. Most pathways decreased to varying degrees following fermentation. This decrease may be attributed to a reduction in microbial diversity and abundance after fermentation, which leads to a relative decrease in metabolic pathways. Alternatively, this may be due to the completion of many microbial pathways during fermentation, resulting in decreased enrichment of related metabolic pathways in the final FYM as the metabolic activities of LAB were completed. Our study revealed that certain metabolic pathways were more enriched in Kangding than in Litang, likely due to Kangding's lower altitude and more favorable climatic conditions for the growth and reproduction of LAB. The diversity and richness of microorganisms in Kangding were higher than those in Litang. Consequently, most metabolic pathways were enriched in Kangding, which is consistent with the microbial diversity results. One of the core metabolic functions of LAB is the synthesis and decomposition of amino acids. Proteins are metabolized into peptides and free amino acids, which contribute not only to basic taste but also to flavor compound precursors of flavor compounds (Ramalingam et al., 2019). Similarly, unsaturated fatty acids in milk undergo oxidation to produce hydroperoxides, which decompose into volatile lipids and oxidation products, such as aldehydes, alcohols, and ketones, further shaping the flavor profile (Shahidi & Hossain, 2022).

Microorganisms metabolize nutrients to produce bacteriocins, lactic acid, and other metabolites, thereby inhibiting or promoting the growth of other microorganisms (Ivey et al., 2013). For instance, our study in Section 3.6, revealed that *L. delbrueckii* was negatively correlated with *S. thermophilus*, which is consistent with the findings of Ding et al. (2022). Previous studies have reported that *Lactobacillus* and *Streptococcus* are negatively correlated in traditional fermented milk (Zhong et al., 2016). While it is accepted that they have a synergistic effect on milk fermentation, the present study suggests that they may have a competitive relationship and eventually reach dynamic equilibrium in FYM. Differential metabolic pathways and microbiota (at the species level) correlation analysis between YM and FYM from the two regions demonstrated that *L. delbrueckii* and *S. thermophilus* were negatively correlated with many pathways. This may be because of their inhibitory effects on the occurrence of pathways. Different microbiota were positively correlated with the metabolic pathways. This may be because the enzymes and substrates produced by the microbiota promote the synthesis of these pathways, and certain metabolites produced by these pathways may promote the growth of microorganisms.

## 5 Conclusion

This study analyzed the microbiota, bacteriophages, and metabolic pathways of yak milk and fermented yak milk from Kangding and Litang in Ganzi using shotgun metagenomic sequencing. We also examined the differences in microbiota between raw and fermented milk from different animals. The results revealed the microbiota composition before and after fermentation of yak milk in both Litang and Kangding. LAB (such as *L. delbrueckii* and *S. thermophilus*) were enriched in fermented yak milk. Compared to Litang, Kangding, which is characterized by a lower altitude and higher average temperature, exhibited greater microbial diversity and a lower relative abundance of genera such as *Lactobacillus*. This may have a positive impact on the taste and flavor of FYM. Comparative analyses of microbiota between raw and fermented milk from different animal sources showed that the microbiota of Bactrian camel milk and yak milk were more similar, with the dominant species being *A. johnsonii* and *P.* unclassified; *L. helveticus* became the absolute dominant species in Koumiss and naturally fermented camel milk. The microbiome of FYM was different from that of the others, with the dominant species being *S. thermophilus* and *L. delbrueckii*. Therefore, FYM may be a potential source of new strains for use in commercial starter cultures. The bacteriophage results indicated that the hosts were primarily harmful microorganisms, and the bacteriophage diversity decreased after fermentation. This indirectly indicates that the fermentation process reduced the number of harmful microorganisms and improved the safety of FYM. Metabolic pathway analysis revealed that carbohydrates, amino acids, fats, and purine metabolism (e.g., UDP-glucose-derived O-antigen building block biosynthesis, L-ornithine biosynthesis, and CDP-diacylglycerol biosynthesis II) were enriched in yak milk, which affected the flavor and sensory properties of fermented yak milk. This study provides valuable insights into the microbial resources of naturally fermented yak milk in the Ganzi region, particularly beneficial microorganisms, and highlights the impact of environmental factors on microbial diversity and fermentation performance. These findings can inform the optimization of fermentation strategies and improve the quality of naturally fermented yak dairy products.

## Funding

National Center of Technology Innovation for Dairy (Grant No. 2023-QNRC-5), Natural Science Foundation of Inner Mongolia, China (Grant No. 2024LHMS03043), and 10.13039/501100001809National Natural Science Foundation of China (Grant No. 32261143729).

## CRediT authorship contribution statement

**Jie Zhang:** Writing – review & editing, Writing – original draft, Data curation. **Yangbo Jiao:** Writing – review & editing, Investigation. **Kaiyang Liu:** Writing – review & editing, Investigation. **Wenyou Situ:** Investigation. **Bilige Menghe:** Supervision, Resources, Project administration. **Yongfu Chen:** Resources, Project administration, Funding acquisition. **Musu Zha:** Writing – review & editing, Resources, Project administration, Funding acquisition.

## Declaration of competing interest

The authors declare that they have no known competing financial interests or personal relationships that could have influenced the work reported in this study.

## Data Availability

Data will be made available on request.

## Appendix A Supplementary data

Supplementary material 1 Metabolic pathways and Spearman correlation analysis. Supplementary material 1

Supplementary material 2 Information of yak milk samples and statistics of sequencing results. Supplementary material 2

Supplementary material 3 Microbial results of four groups (at the phylum, genus and species level). Supplementary material 3

Supplementary material 4 Metabolic pathway results of four groups Supplementary material 4
