# Supplementary material for: Characterizing microbial diversity and metabolic pathways in yak milk and fermented yak milk based on metagenomics: A study from Ganzi Tibetan autonomous prefecture
Source: Food Chem X. 2025 Jan 18;25:102198. doi: 10.1016/j.fochx.2025.102198 (PMC11791328; doi:10.1016/j.fochx.2025.102198)
Supplement: Supplementary material 1 — Metabolic pathways and Spearman correlation analysis. [file mmc1.docx]

Supplementary Figure

**Fig. S1** Analysis of metabolic pathways differences between yak milk and fermented yak milk form different regions (A). Differences between yak milk and fermented yak milk at Litang (B). Differences between yak milk and fermented yak milk at Kangding (C). Differences between Litang and Kangding of fermented yak milk (D).

**Fig. S2** Microbial association network with relative abundance greater than 1% at the species level based on Spearman’s correlation analysis (A). Correlation analysis between differential metabolic pathways and differential strains at the species (B).


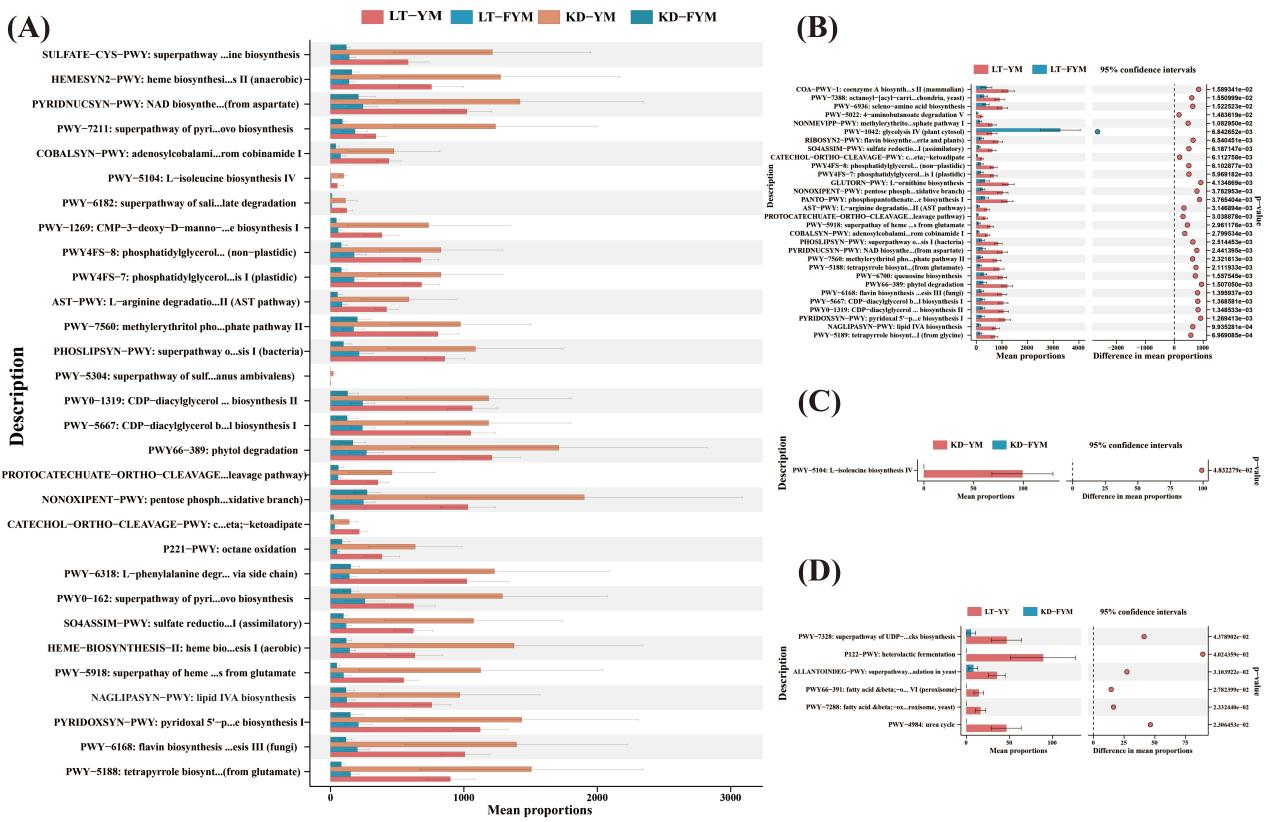


Fig. S1. Analysis of metabolic pathways differences between yak milk and fermented yak milk form different regions (A). Differences between yak milk and fermented yak milk at Litang (B). Differences between yak milk and fermented yak milk at Kangding (C). Differences between Litang and Kangding of fermented yak milk (D).


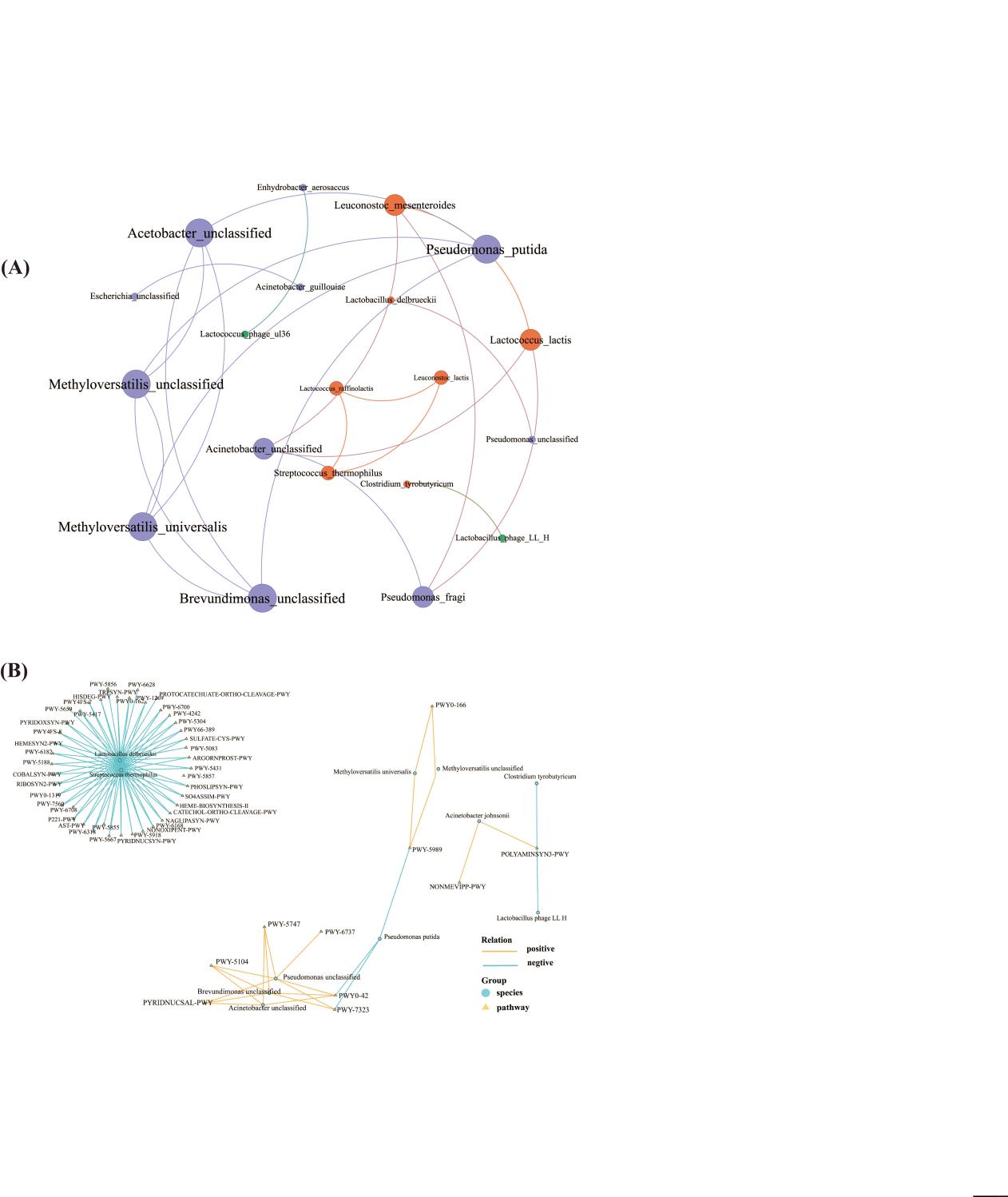


Fig. S2. Microbial association network with relative abundance greater than 1% at the species level based on Spearman’s correlation analysis. Nodes are colored by phylum. Orange nodes represents *Proteobacteria*, purple nodes represent *Firmicutes*, and green nodes represent *Viruses* noname. Orange and dark red edges represent positive correlations, purple edges represent negative correlations, *Enhydrobacter aerosaccus* and *Lactococcus phage ul36* were negatively correlated, *Clostridium tyrobutyricum* and *Lactobacillus phage LL H* were positively correlated. The size of the node in the figure represents the species abundance, and the larger the abundance value, the larger the node (A). Correlation analysis between differential metabolic pathways and differential strains at the species. Line color represented positive and negative correlations (*P* < 0.05), and circles represent species, triangles represent pathways (B).
