# Supplementary material for: Characterizing microbial diversity and metabolic pathways in yak milk and fermented yak milk based on metagenomics: A study from Ganzi Tibetan autonomous prefecture
Source: Food Chem X. 2025 Jan 18;25:102198. doi: 10.1016/j.fochx.2025.102198 (PMC11791328; doi:10.1016/j.fochx.2025.102198)
Supplement: Supplementary material 2 — Information of yak milk samples and statistics of sequencing results. [file mmc2.docx]

Supplementary Table

Table S1 Information about yak milk and fermented yak milk collected in different regions

| **Sample** | | | **Sampling location** | **Altitude** | | **Latitude and longitude** |
| --- | --- | --- | --- | --- | --- | --- |
| **Group** | **YM-number** | **FYM-number** |  | **Original altitude** | **Average altitude** |  |
| LT | SC91 | SC92 | Heranseba Cun, Heni Xiang, Litang Xian, Ganzi Tibetan Autonomous Prefecture, Sichuan Province, China | 3941.9m | 3848.26±126.78^b^ | N30°12'1''; E99°46'25'' |
|  | SC94 | SC95 | Heranseba Cun, Heni Xiang, Litang Xian, Ganzi Tibetan Autonomous Prefecture, Sichuan Province, China | 3941.9m |  | N30°12'1''; E99°46'25'' |
|  | SC99 | SC101 | Heranseba Cun, Heni Xiang, Litang Xian, Ganzi Tibetan Autonomous Prefecture, Sichuan Province, China | 3941.9m |  | N30°12'1''; E99°46'25'' |
|  | SC103 | SC102 | Heranseba Cun, Heni Xiang, Litang Xian, Ganzi Tibetan Autonomous Prefecture, Sichuan Province, China | 3941.9m |  | N30°12'1''; E99°46'25'' |
|  | SC106 | SC107 | Heranseba Cun, Heni Xiang, Litang Xian, Ganzi Tibetan Autonomous Prefecture, Sichuan Province, China | 3941.9m |  | N30°12'1''; E99°46'25'' |
|  | SC109 | SC108 | Heranseba Cun, Heni Xiang, Litang Xian, Ganzi Tibetan Autonomous Prefecture, Sichuan Province, China | 3941.9m |  | N30°12'1''; E99°46'25'' |
|  | SC111 | SC112 | Sage cun, Benge Xiang, Litang Xian, Ganzi Tibetan Autonomous Prefecture, Sichuan province, China | 3684.4m |  | N29°56'46''; E100°17'52'' |
|  | SC114 | SC113 | Sage cun, Benge Xiang, Litang Xian, Ganzi Tibetan Autonomous Prefecture, Sichuan province, China | 3684.4m |  | N29°56'46''; E100°17'52'' |
|  | SC115 | SC116 | Sage cun, Benge Xiang, Litang Xian, Ganzi Tibetan Autonomous Prefecture, Sichuan province, China | 3684.4m |  | N29°56'46''; E100°17'52'' |
|  | SC118 | SC117 | Sage cun, Benge Xiang, Litang Xian, Ganzi Tibetan Autonomous Prefecture, Sichuan province, China | 3684.4m |  | N29°56'46''; E100°17'52'' |
|  | SC122 | SC123 | Sage cun, Benge Xiang, Litang Xian, Ganzi Tibetan Autonomous Prefecture, Sichuan province, China | 3941.9m |  | N29°55'11''; E100°18'2'' |
| KD | SC43 | SC44 | Jiangba Cun, Tagong Zhen, , Kangding City, Ganzi Tibetan Autonomous Prefecture, Sichuan Province, China | 3845.4m | 3765.33±64.64^a^ | N30°10'31''; E101°42'22'' |
|  | SC45 | SC46 | Jiangba Cun, Tagong Zhen, , Kangding City, Ganzi Tibetan Autonomous Prefecture, Sichuan Province, China | 3771.1m |  | N30°11'25''; E101°40'7'' |
|  | SC57 | SC56 | Shangbaisangyi cun, Xinduqiao Zhen, Kangding City, Ganzi Tibetan Autonomous Prefecture, Sichuan province, China | 3675m |  | N30°11'20''; E101°30'26'' |
|  | SC80 | SC81 | Xiamalong cun, Tagong Zhen, Kangding City, Ganzi Tibetan Autonomous Prefecture, Sichuan province, China | 3769.8m |  | N30°18'12''; E101°33'40'' |

Note: LT represents Litang Xian, Ganzi Tibetan Autonomous Prefecture, Sichuan Province, China; KD represents Kangding City, Ganzi Tibetan Autonomous Prefecture, Sichuan Province, China; YM represents yak milk; FYM represents fermented yak milk. Data are analyzed using a Two-Sample T-Test. The value of average altitude is expressed as mean ± SD. ^a-b^ Different letters indicate significant differences (*P* < 0.05).

Table S2 Statistical table of samples sequencing results

| **Sample** | | **Raw Reads** | **Clean Reads** | **Raw Base(G)** | **Clean Base**  **(G)** | **Percent in raw reads**  **（%）** | **Q20(%)** | **Q30(%)** | **GC content**  **(%)** |
| --- | --- | --- | --- | --- | --- | --- | --- | --- | --- |
| **Group** | **Number** |  |  |  |  |  |  |  |  |
| LT-YM | SC91 | 46100552 | 45504322 | 6.92 | 6.83 | 98.71 | 97.07 | 92.67 | 48.15 |
|  | SC94 | 49527658 | 49075098 | 7.43 | 7.36 | 99.09 | 97.07 | 92.48 | 48.23 |
|  | SC99 | 49620654 | 49275768 | 7.44 | 7.39 | 99.3 | 97.46 | 93.16 | 43.38 |
|  | SC103 | 49311360 | 48863446 | 7.4 | 7.33 | 99.09 | 97.28 | 92.84 | 50.06 |
|  | SC106 | 44976880 | 44193754 | 6.75 | 6.63 | 98.26 | 96.98 | 92.47 | 44.59 |
|  | SC109 | 44462912 | 44153332 | 6.67 | 6.62 | 99.3 | 97.55 | 93.28 | 41.03 |
|  | SC111 | 46374998 | 45705728 | 6.96 | 6.86 | 98.56 | 97.1 | 92.78 | 45.99 |
|  | SC114 | 44185492 | 43767694 | 6.63 | 6.57 | 99.05 | 97.75 | 93.92 | 49.69 |
|  | SC115 | 47137566 | 46854944 | 7.07 | 7.03 | 99.4 | 97.61 | 93.37 | 44.29 |
|  | SC118 | 45216536 | 45098562 | 6.78 | 6.76 | 99.74 | 97.81 | 93.69 | 45.71 |
|  | SC122 | 47982736 | 47651282 | 7.2 | 7.15 | 99.31 | 97.5 | 93.18 | 44.52 |
| LT-FYM | SC92 | 46443862 | 45777652 | 6.97 | 6.87 | 98.57 | 97.1 | 92.59 | 44.88 |
|  | SC95 | 45567948 | 45400722 | 6.84 | 6.81 | 99.63 | 97.81 | 93.71 | 46.97 |
|  | SC101 | 47124386 | 46885252 | 7.07 | 7.03 | 99.49 | 97.68 | 93.56 | 47.6 |
|  | SC102 | 49931374 | 49808818 | 7.49 | 7.47 | 99.75 | 97.74 | 93.51 | 46.18 |
|  | SC107 | 43086278 | 42983516 | 6.46 | 6.45 | 99.76 | 97.9 | 93.68 | 37.99 |
|  | SC108 | 49870652 | 49756168 | 7.48 | 7.46 | 99.77 | 97.91 | 93.74 | 37.8 |
|  | SC112 | 52825974 | 51767572 | 7.92 | 7.77 | 98 | 96.87 | 92.5 | 44.65 |
|  | SC113 | 47186900 | 46993932 | 7.08 | 7.05 | 99.59 | 97.64 | 93.39 | 44.28 |
|  | SC116 | 50067560 | 49947426 | 7.51 | 7.49 | 99.76 | 97.63 | 93.18 | 40.67 |
|  | SC117 | 46804292 | 46687052 | 7.02 | 7 | 99.75 | 97.76 | 93.56 | 45.51 |
|  | SC123 | 44631782 | 44457270 | 6.69 | 6.67 | 99.61 | 97.73 | 93.48 | 45.56 |
| KD-YM | SC43 | 65799436 | 65089086 | 9.87 | 9.76 | 98.92 | 97.38 | 93.36 | 51.56 |
|  | SC45 | 51066706 | 50472072 | 7.66 | 7.57 | 98.84 | 97.29 | 93.14 | 44.14 |
|  | SC57 | 52610872 | 52024672 | 7.89 | 7.8 | 98.89 | 97.29 | 93.19 | 45.46 |
|  | SC80 | 62252462 | 61378472 | 9.34 | 9.21 | 98.6 | 97.02 | 92.64 | 45.03 |
| KD-FYM | SC44 | 43817250 | 43687518 | 6.57 | 6.55 | 99.7 | 97.99 | 94.12 | 48.18 |
|  | SC46 | 45584172 | 45424826 | 6.84 | 6.81 | 99.65 | 97.75 | 93.57 | 46.46 |
|  | SC56 | 44969092 | 44823722 | 6.75 | 6.72 | 99.68 | 97.62 | 93.28 | 46.38 |
|  | SC81 | 44607300 | 44432948 | 6.69 | 6.66 | 99.61 | 97.78 | 93.67 | 47.33 |
